# Supplementary material for: Evaluating the psychosocial status of BC children and youth during the COVID-19 pandemic: A MyHEARTSMAP cross-sectional study
Source: PLoS One. 2023 Mar 31;18(3):e0281083. doi: 10.1371/journal.pone.0281083 (PMC10065280; doi:10.1371/journal.pone.0281083)

**Supplementary Information**

**Methods**

To assess for the effect of responder, separate proportional odds models were generating to include all youth-completed assessments (N = 225) and all guardian-completed assessments (N = 381) separately. For the original model, if both youth- and guardian-completed assessments were available, only the more severe rating was included. Due to this difference in samples it is difficult to compare these results directly to the primary analysis. Due to a small sample size in the severe category, it was required to combine the moderate and severe groups to allow the models to converge.

**Results**

Using proportional odds models, the likelihood of reporting greater severity of difficulty was examined for the psychiatry, social, and youth health domains for all youth- and guardian-completed assessments (see Supplementary Figures 1 and 2 respectively). For youth-completed assessments, older age was associated with greater severity across the psychiatry (OR = 1.22, 95% CI = 1.09, 1.38), social (OR = 1.19, 95% CI = 1.05, 1.35), and youth health (OR = 1.18, 95% CI = 1.04, 1.35) domains. Girls were more likely to report greater difficulty in the psychiatry (OR = 1.96, 95% CI = 1.11, 3.50), social (OR = 2.28, 95% CI = 1.54, 5.34), and youth health (OR = 2.19, 95% CI = 1.21, 4.03) domains. Youth with nonbinary or questioning gender identity experience greater severity in the youth health domain (OR = 5.90, 95% CI = 1.05, 34.14). Children and adolescents are Black or Indigenous were more likely to experience greater severity in the psychiatry (OR = 11.01, 95% CI = 1.92, 86.51) domain. Youth who attended full-time homeschooling or virtual/remote schooling were more likely to report greater difficulty in the social (OR = 3.94, 95% CI = 1.50, 10.74) and youth health (OR = 2.76, 95% CI = 1.12, 6.85) domains. Children living in the Interior Health Authority may be less likely to experience greater social difficulty (OR = 0.21, 95% CI = 0.07, 0.63) compared to children living in the Vancouver Coastal Health Authority. The role of all other factors including neighbourhood income, and guardian unemployment is uncertain, as confidence intervals spanned both increases and decreases in MyHEARTSMAP severity.

For guardian-completed assessments, older age was associated with greater severity across the psychiatry (OR = 1.20, 95% CI = 1.12, 1.29), social (OR = 1.18, 95% CI = 1.04, 1.34), and youth health (OR = 1.15, 95% CI = 1.07, 1.23) domains. Girls were more likely to experience greater social difficulty (OR = 2.99, 95% CI = 1.61, 5.69) and youth with nonbinary or questioning gender identity were more likely to experience greater severity in the psychiatry domain (OR = 4.8, 95% CI = 1.22, 18.44). Children not attending any school or formal education program when it would otherwise be in session were more likely to experience greater difficulty in the psychiatry (OR = 2.72, 95% CI = 1.36, 5.43) and youth health (OR = 2.04, 95% CI = 1.07, 3.90) and youth being homeschooled or attending school full-time virtually or remote were more likely to experience greater social difficulty (OR = 3.96, 95% CI = 1.46, 11.13). Youth living in the Interior Health Authority may experience less severe social difficulty (OR = 0.22, 95% CI = 0.07, 0.66) compared to youth in Vancouver. The role of all other factors including ethnicity, neighbourhood income, and guardian unemployment is uncertain.

**Interpretation**

The results of the proportional odds models for each responder group generally support the findings of the larger sample, with age, gender, and school status being notable factors. Children who are Black or Indigenous may benefit from anti-racist and culturally informed community mental health resources. The Interior Health Authority region experienced relatively lower case counts of COVID-19 compared to other regions throughout the period of recruitment and this may explain why child and guardians identified less social difficulty during this time-period.

**Tables and Figures**

S1 Fig: Organisation of 10 psychosocial sections into four domains used for resource recommendation by MyHEARTSMAP assessment.


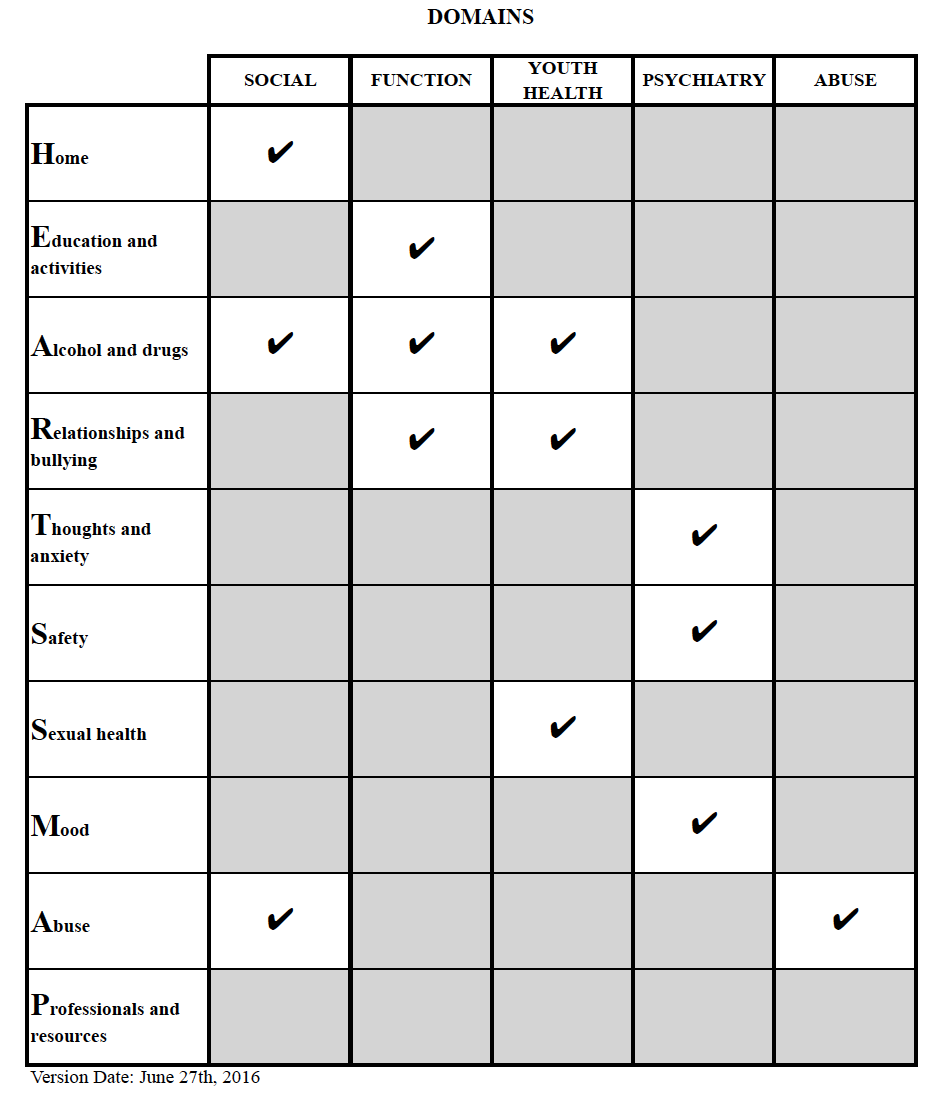


S1 Table: List of community organizations and groups who assisted with study recruitment through distribution of recruitment materials to their networks

| **Youth Organizations** |
| --- |
| Broadway Youth Resource Centre |
| Covenant House |
| Gastown Vocational Services |
| Take a Hike |
| Vancouver Cadets |
|  |
| **Community Groups** |
| Association of Neighbourhood Houses BC |
| Britannia Community Services Centre |
| Burnside Gorge Community Association |
| Frog Hollow Neighbourhood House |
| Marpole Oakridge Family Place |
| Oaklands Community Association |
| YMCA Okanagan |
| Tri-City Family Place |
|  |
| **Mental Health Organizations** |
| Adam’s Apple Foundation |
| Compass Mental Health |
| Encompass Support Services BC |
| Jack.org |
| Nanaimo Youth Services |
| Safe Online |
| Youth Space |
|  |
| **Children’s Advocacy Organizations** |
| First Call: BC Child and Youth Advocacy Coalition |
| Oak Child and Youth Advocacy Centre |
| SKY |
| Treehouse Vancouver |
|  |
| **Indigenous Groups** |
| Aboriginal Supported Child Development |
| Denisiqi Services Society |
| KUU-US Crisis Response Services |
| Indigenous Youth Wellness Group |
| Ookna Kane Friendship Centre |
| Sechelt Indian Band Child & Family Services |
| Usma Nuu-chah-nulth Family & Child Services |
| Waclay Friendship Centre Society |
|  |
| **Other** |
| Mainland BC Military Family Resource Centre (Canadian Armed Forces) |
| SOLOS – Safe Online Outreach Society |

S2 Table: Comparison of demographic variables and risk factors for individuals participated in our study and completed the MyHEARTSMAP assessment at baseline compared to those who did not.

| **Factors** | **Did not complete**  (N = 124)* | **Completed**  (N = 425)* | **Standard mean difference** | **p-value** |
| --- | --- | --- | --- | --- |
| **Age (mean (SD))** | 11.36 (3.39) | 10.84 (3.30) | 0.157 | 0.121 |
| **Ethnicity (N (%))** |  |  | 0.358 | 0.071 |
| Other terms combined | 8 (9.0) | 17 (4.1) |  |  |
| Asian | 4 (4.5) | 27 (6.6) |  |  |
| Multiethnic | 18 (20.2) | 68(16.6) |  |  |
| White | 59 (66.3) | 298 (72.7) |  |  |
| **Gender** |  |  | 0.187 | 0.203 |
| Other terms combined | 4 (4.4) | 9 (2.1) |  |  |
| Girl/Young woman | 51 (56.0) | 211 (49.6) |  |  |
| Boy/Young man | 36 (39.6) | 205 (48.2) |  |  |
| **Income in area (mean (SD))** | 50453.42 (12514.22) | 52666.28 (12953.55) | 0.137 | 0.174 |
| **School status** |  |  | 0.129 | 0.282 |
| Homeschool and virtual/remote schooling | 13 (14.3) | 65 (15.3) |  |  |
| Hybrid or part time in-person schooling | 11 (12.1) | 64 (15.1) |  |  |
| No school or formal education program | 10 (11.0) | 55 (13.0) |  |  |
| Summer holiday | 17 (18.7) | 39 (9.2) |  |  |
| Full time in-person school | 40 (44.0) | 201 (47.4) |  |  |
| **Health status** |  |  | 0.172 | 0.293 |
| No known medical conditions | 72 (79.1) | 364 (85.6) |  |  |
| Chronic but stable medical condition | 16 (17.6) | 52 (12.2) |  |  |
| Chronic with frequent or regular healthcare needs | 3 (3.3) | 9 (2.1) |  |  |
| **Mental health status** |  |  | 0.281 | 0.039 |
| No known mental health conditions | 60 (65.9) | 333 (78.4) |  |  |
| Chronic but stable mental health condition | 25 (27.5) | 72 (16.9) |  |  |
| Chronic with frequent or regular exacerbations | 6 (6.6) | 20 (4.7) |  |  |
| **Employment** |  |  | 0.440 | 0.006 |

*Category totals may not reflect sample size due to missing data

S3 Table: Results from multivariable proportional odds model indicating the odds of increased severity score (0-7) in the psychiatry, social, and youth health domains.

| **Factors** | **Psychiatry**  OR (95% CI) | **Social**  OR (95% CI) | **Youth Health**  OR (95% CI) |
| --- | --- | --- | --- |
| **Age (per year increase)** | 1.29 (1.20, 1.39) | 1.23 (1.15, 1.33) | 1.18 (1.10, 1.26) |
| **Ethnicity** |  |  |  |
| Other terms combined | 1.22 (0.42, 3.47) | 0.73 (0.25, 2.14) | 1.10 (0.38, 3.03) |
| Asian | 0.72 (0.31, 1.66) | 1.30 (0.55, 3.12) | 0.50 (0.20, 1.19) |
| Multiethnic | 1.36 (0.77, 2.38) | 1.74 (0.97, 3.14) | 0.96 (0.55, 1.67) |
| White | - | - | - |
| **Gender** |  |  |  |
| Other terms combined | 4.19 (1.07, 16.13) | 3.76 (0.91, 15.97) | 5.03 (1.39, 18.73) |
| Girl/Young Woman | 1.31 (0.86, 2.01) | 2.03 (1.32, 3.16) | 1.08 (0.71, 1.64) |
| Boy/Young Man | - | - | - |
| **Health authority** |  |  |  |
| Fraser | 1.46 (0.86, 2.50) | 1.21 (0.69, 2.12) | 1.07 (0.62, 1.84) |
| Interior | 0.73 (0.33, 1.62) | 0.69 (0.31, 1.53) | 0.88 (0.41, 1.90) |
| Island Health | 0.81 (0.43, 1.51) | 1.36 (0.71, 2.61) | 0.74 (0.39, 1.39) |
| Northern | 0.55 (0.23, 1.30) | 0.62 (0.26, 1.50) | 0.89 (0.36, 2.13) |
| Vancouver Coastal | - | - | - |
| **Average income in area (per $5000)** | 0.90 (0.82, 0.98) | 0.91 (0.83, 0.99) | 0.91 (0.82, 0.99) |
| **School status** |  |  |  |
| Homeschool and Virtual/Remote Schooling | 0.99 (0.54, 1.81) | 1.59 (0.85, 3.01) | 2.10 (1.17, 3.77) |
| Hybrid or Part Time In-Person Schooling | 1.29 (0.69, 2.41) | 0.78 (0.41, 1.51) | 1.27 (0.68, 2.34) |
| No School or Formal Education Program | 2.30 (1.20, 4.42) | 0.92 (0.47, 1.81) | 2.10 (1.12, 3.94) |
| Summer Holiday | 0.73 (0.35, 1.52) | 1.12 (0.53, 2.37) | 0.77 (0.35, 1.61) |
| Full Time In-Person School | - | - | - |
| **Guardian unemployed** | 1.23 (0.72, 2.09) | 1.21 (0.70, 2.12) | 1.38 (0.81, 2.34) |

*OR = Odds Ratio; CI = Confidence Interval

- denotes reference

S2 Fig: Results from multivariable proportional odds model indicating the odds of increased severity score (0-7) in the psychiatry, social, and youth health domains for all youth-completed assessments.


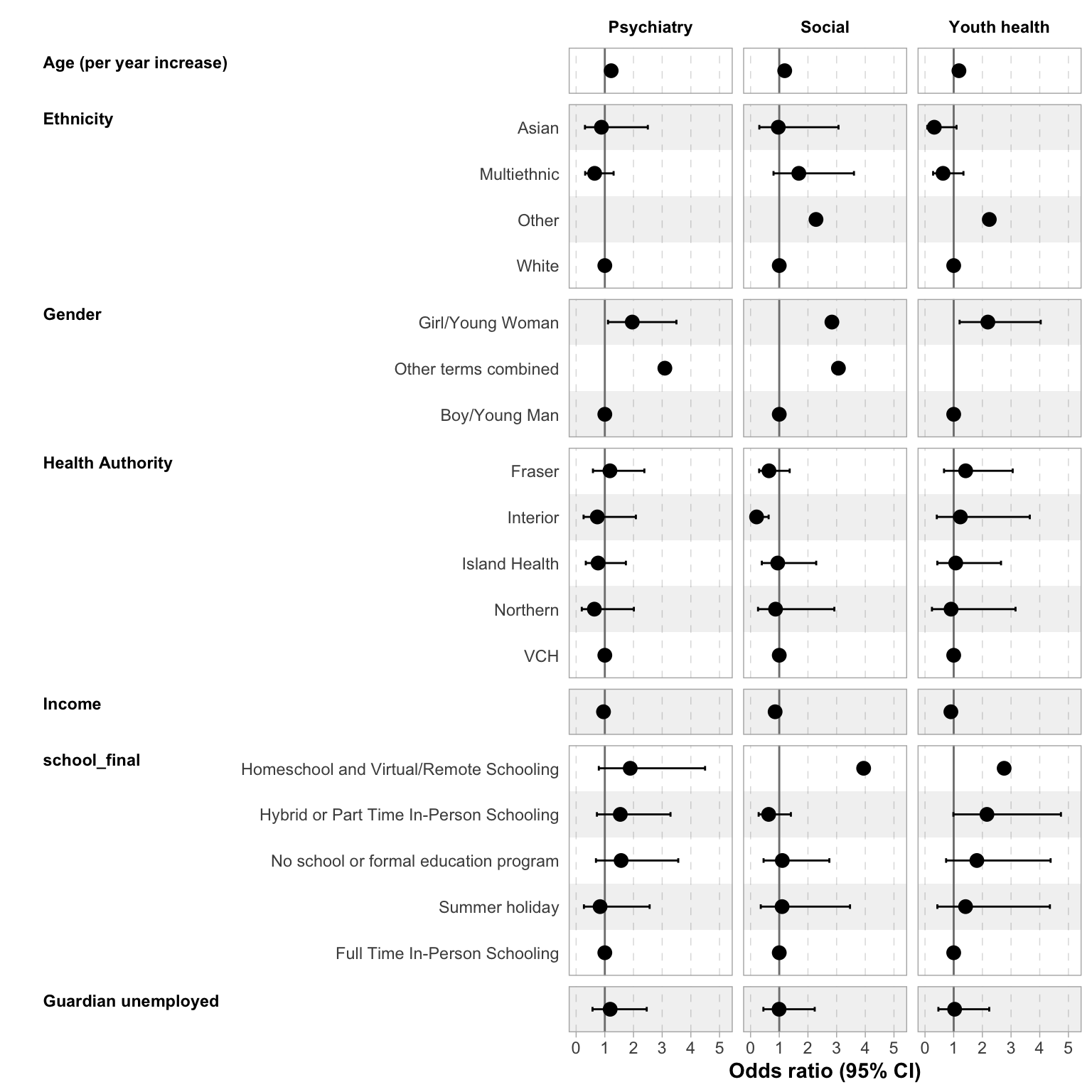


S3 Fig: Results from multivariable proportional odds model indicating the odds of increased severity score (0-7) in the psychiatry, social, and youth health domains for all guardian-completed assessments.


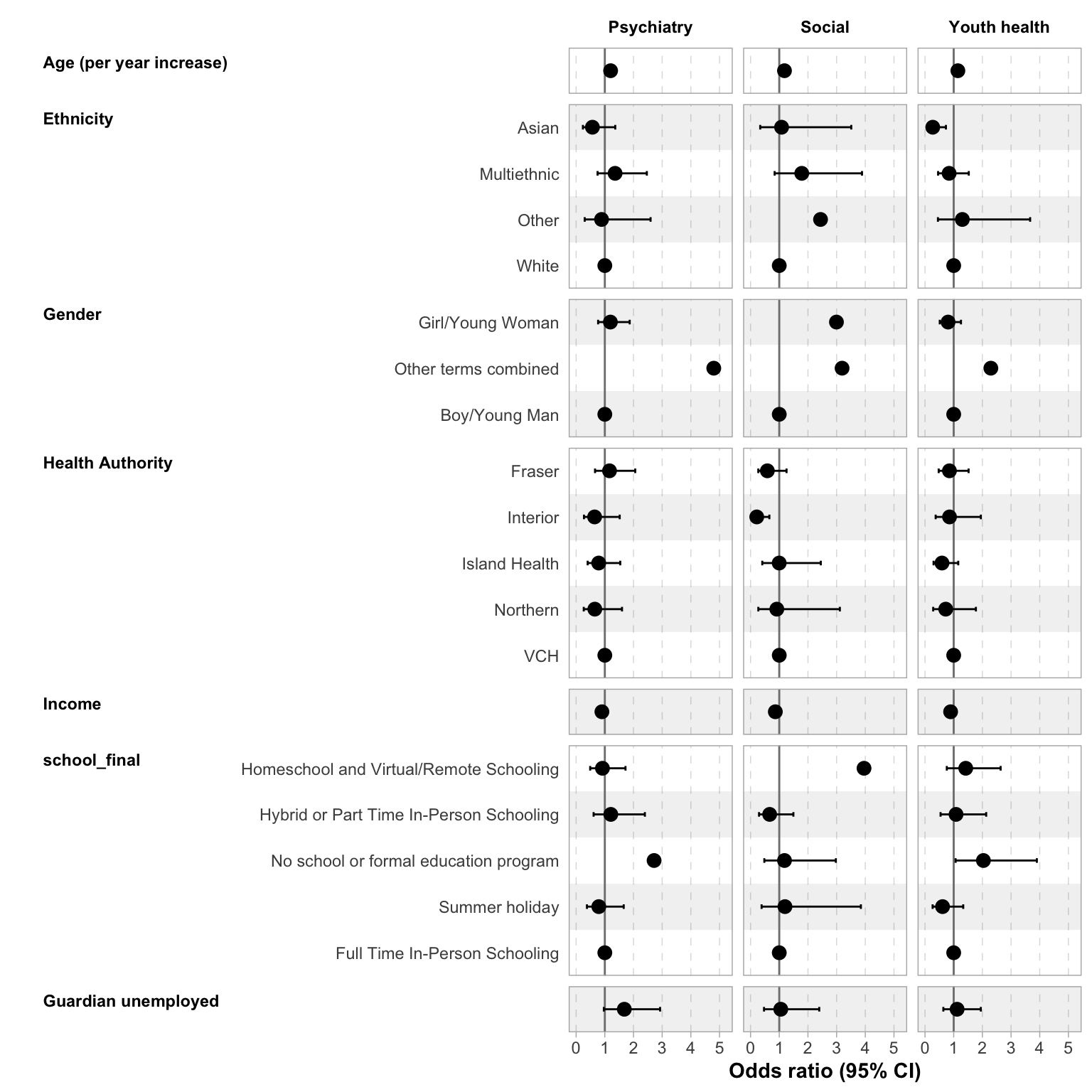

Supplement: S2 File — (DOCX) [file pone.0281083.s002.docx]
