# Supplementary figures and images for: Evaluating the psychosocial status of BC children and youth during the COVID-19 pandemic: A MyHEARTSMAP cross-sectional study
Source: PLoS One. 2023 Mar 31;18(3):e0281083. doi: 10.1371/journal.pone.0281083 (PMC10065280; doi:10.1371/journal.pone.0281083)

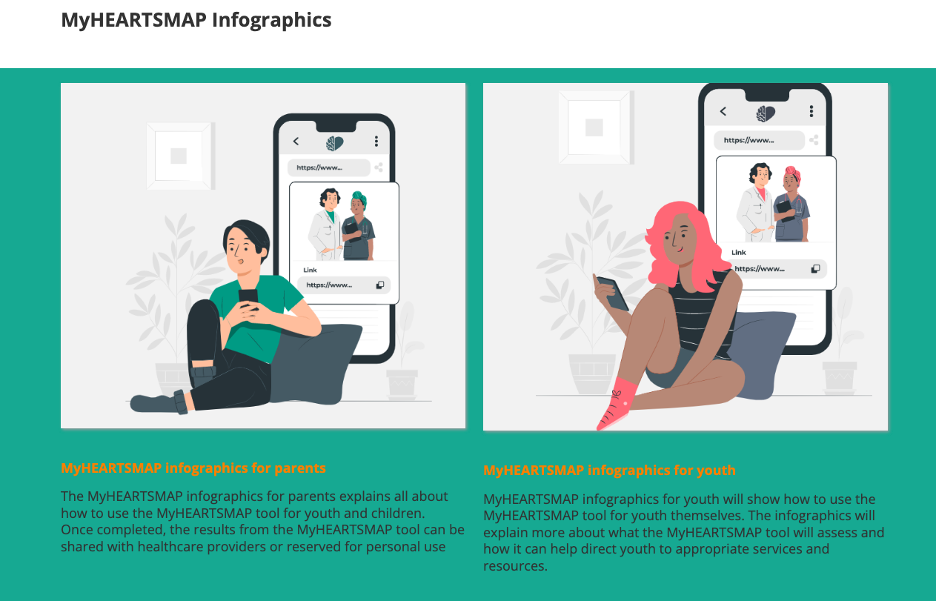

Supplement: S1 Fig — (TIF) [file pone.0281083.s003.tif]

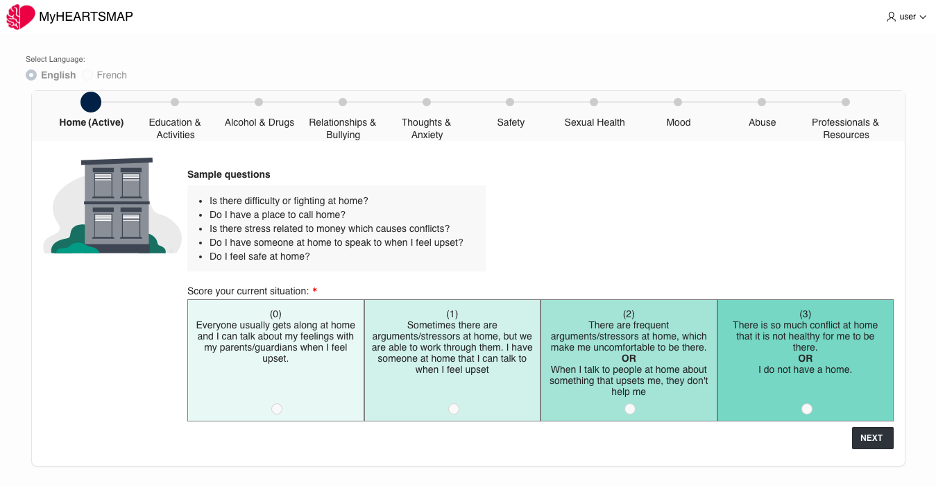

Supplement: S2 Fig — (TIF) [file pone.0281083.s004.tif]

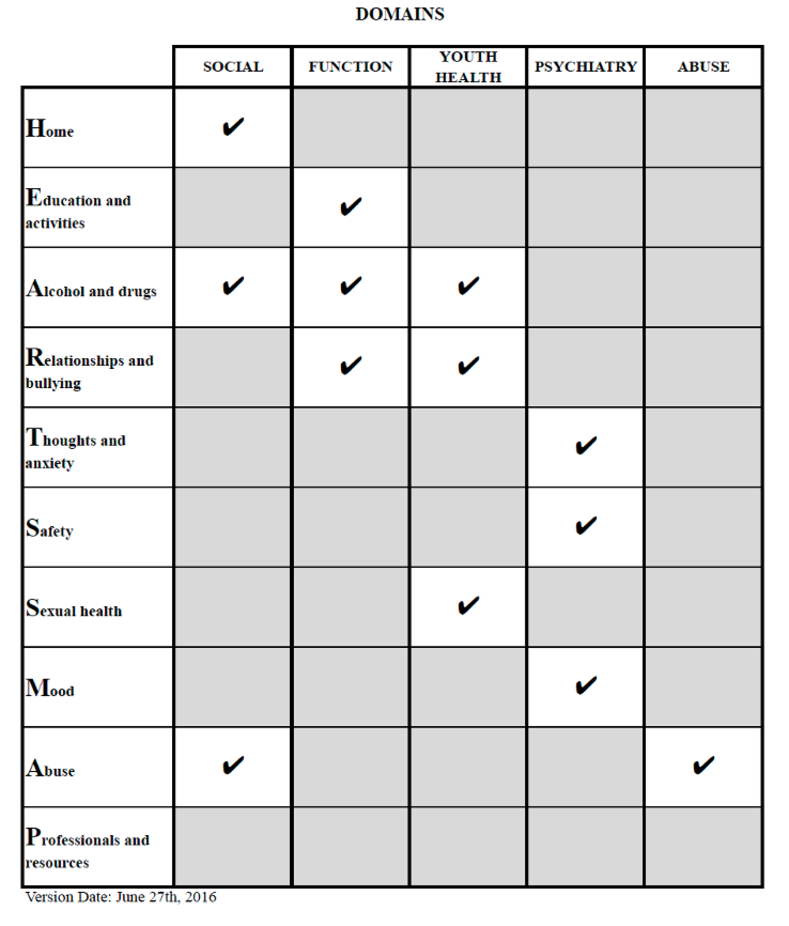

Supplement: S3 Fig — (TIF) [file pone.0281083.s005.tif]

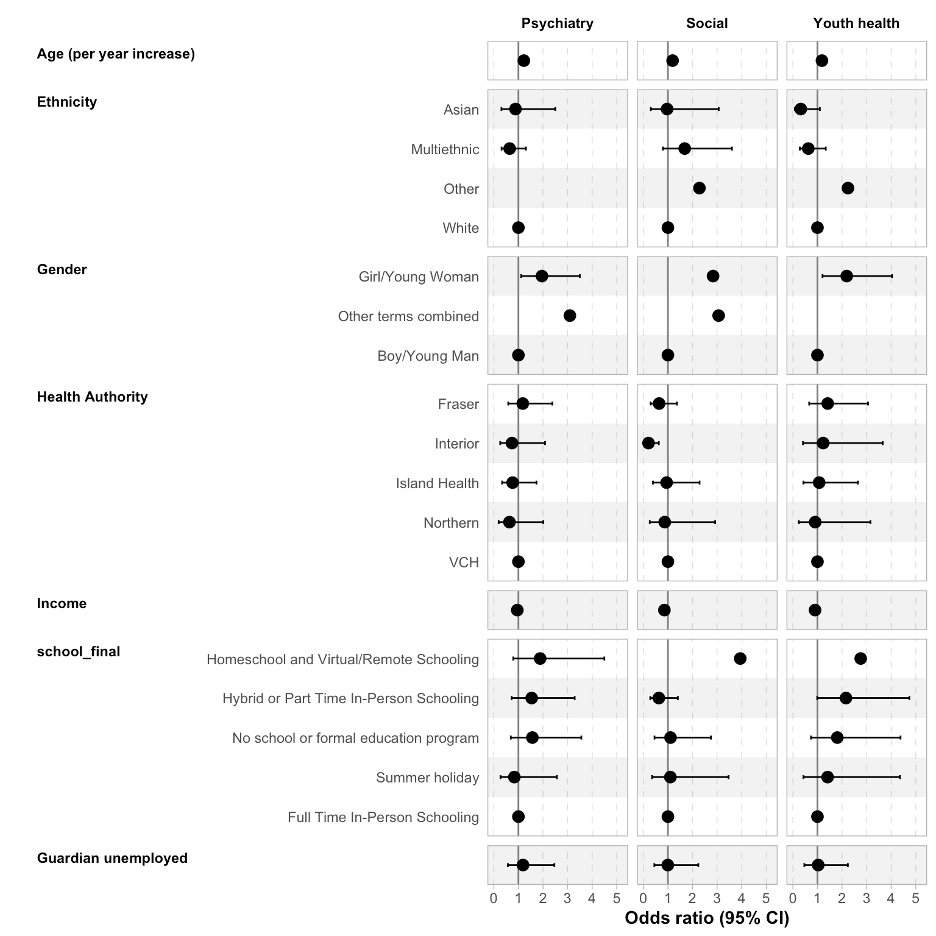

Supplement: S4 Fig — (TIF) [file pone.0281083.s006.tif]

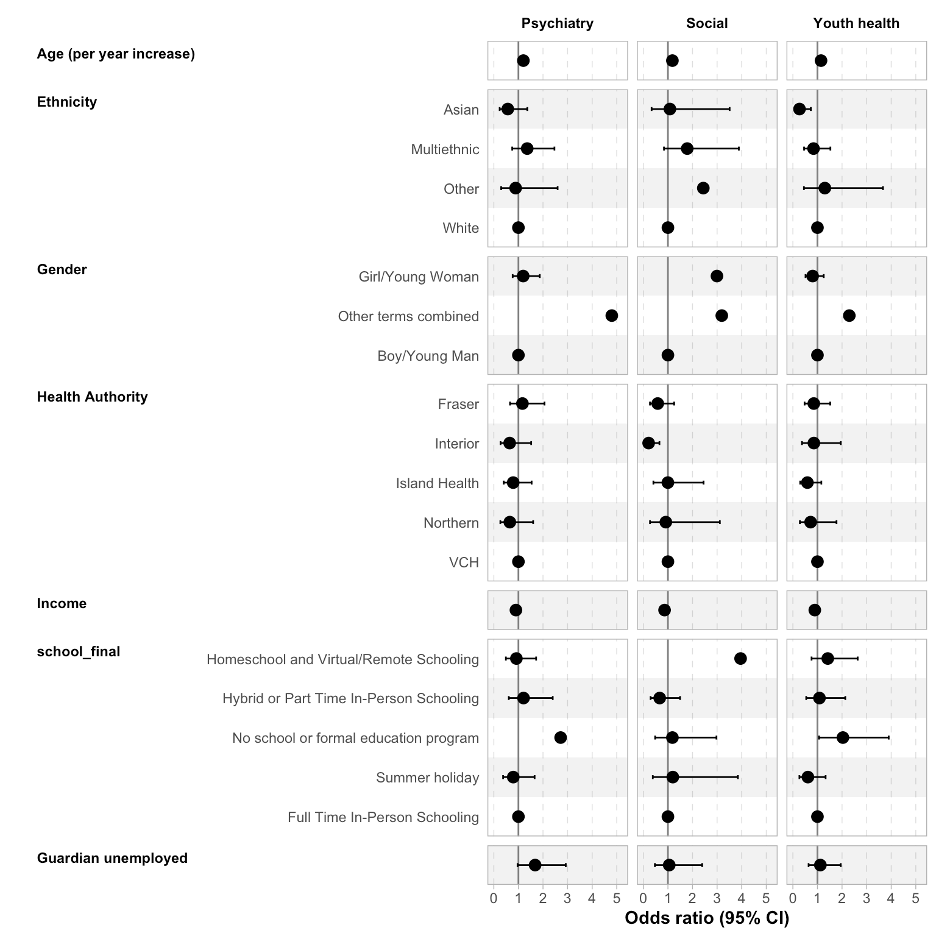

Supplement: S5 Fig — (TIF) [file pone.0281083.s007.tif]
