# Supplementary material for: Evaluating the psychosocial status of BC children and youth during the COVID-19 pandemic: A MyHEARTSMAP cross-sectional study
Source: PLoS One. 2023 Mar 31;18(3):e0281083. doi: 10.1371/journal.pone.0281083 (PMC10065280; doi:10.1371/journal.pone.0281083)
Supplement: S1 Table — (DOCX) [file pone.0281083.s008.docx]

**S1 Table: List of community organizations and groups who assisted with study recruitment through distribution of recruitment materials to their networks.**

| **Youth Organizations** |
| --- |
| Broadway Youth Resource Centre |
| Covenant House |
| Gastown Vocational Services |
| Take a Hike |
| Vancouver Cadets |
| **Community Groups** |
| Association of Neighbourhood Houses BC |
| Britannia Community Services Centre |
| Burnside Gorge Community Association |
| Frog Hollow Neighbourhood House |
| Marpole Oakridge Family Place |
| Oaklands Community Association |
| YMCA Okanagan |
| Tri-City Family Place |
| **Mental Health Organizations** |
| Adam’s Apple Foundation |
| Compass Mental Health |
| Encompass Support Services BC |
| Jack.org |
| Nanaimo Youth Services |
| Safe Online |
| Youth Space |
| **Children’s Advocacy Organizations** |
| First Call: BC Child and Youth Advocacy Coalition |
| Oak Child and Youth Advocacy Centre |
| SKY |
| Treehouse Vancouver |
| **Indigenous Groups** |
| Aboriginal Supported Child Development |
| Denisiqi Services Society |
| KUU-US Crisis Response Services |
| Indigenous Youth Wellness Group |
| Ookna Kane Friendship Centre |
| Sechelt Indian Band Child & Family Services |
| Usma Nuu-chah-nulth Family & Child Services |
| Waclay Friendship Centre Society |
| **Other** |
| Mainland BC Military Family Resource Centre (Canadian Armed Forces) |
| SOLOS – Safe Online Outreach Society |
