# Supplementary material for: Evaluating the psychosocial status of BC children and youth during the COVID-19 pandemic: A MyHEARTSMAP cross-sectional study
Source: PLoS One. 2023 Mar 31;18(3):e0281083. doi: 10.1371/journal.pone.0281083 (PMC10065280; doi:10.1371/journal.pone.0281083)
Supplement: S2 Table — (DOCX) [file pone.0281083.s009.docx]

**S2 Table: Comparison of demographic variables and risk factors for individuals participated in our study and completed the MyHEARTSMAP assessment at baseline compared to those who did not.**

| **Factors** | **Did not complete**  (N = 124)* | **Completed**  (N = 425)* | **Standard mean difference** | **p-value** |
| --- | --- | --- | --- | --- |
| **Age (mean (SD))** | 11.36 (3.39) | 10.84 (3.30) | 0.157 | 0.121 |
| **Ethnicity (N (%))** |  |  | 0.358 | 0.071 |
| Other terms combined | 8 (9.0) | 17 (4.1) |  |  |
| Asian | 4 (4.5) | 27 (6.6) |  |  |
| Multiethnic | 18 (20.2) | 68(16.6) |  |  |
| White | 59 (66.3) | 298 (72.7) |  |  |
| **Gender** |  |  | 0.187 | 0.203 |
| Other terms combined | 4 (4.4) | 9 (2.1) |  |  |
| Girl/Young woman | 51 (56.0) | 211 (49.6) |  |  |
| Boy/Young man | 36 (39.6) | 205 (48.2) |  |  |
| **Income in area (mean (SD))** | 50453.42 (12514.22) | 52666.28 (12953.55) | 0.137 | 0.174 |
| **School status** |  |  | 0.129 | 0.282 |
| Homeschool and virtual/remote schooling | 13 (14.3) | 65 (15.3) |  |  |
| Hybrid or part time in-person schooling | 11 (12.1) | 64 (15.1) |  |  |
| No school or formal education program | 10 (11.0) | 55 (13.0) |  |  |
| Summer holiday | 17 (18.7) | 39 (9.2) |  |  |
| Full time in-person school | 40 (44.0) | 201 (47.4) |  |  |
| **Health status** |  |  | 0.172 | 0.293 |
| No known medical conditions | 72 (79.1) | 364 (85.6) |  |  |
| Chronic but stable medical condition | 16 (17.6) | 52 (12.2) |  |  |
| Chronic with frequent or regular healthcare needs | 3 (3.3) | 9 (2.1) |  |  |
| **Mental health status** |  |  | 0.281 | 0.039 |
| No known mental health conditions | 60 (65.9) | 333 (78.4) |  |  |
| Chronic but stable mental health condition | 25 (27.5) | 72 (16.9) |  |  |
| Chronic with frequent or regular exacerbations | 6 (6.6) | 20 (4.7) |  |  |
| **Employment** |  |  | 0.440 | 0.006 |

*Category totals may not reflect sample size due to missing data
