# Supplementary material for: Evaluating the psychosocial status of BC children and youth during the COVID-19 pandemic: A MyHEARTSMAP cross-sectional study
Source: PLoS One. 2023 Mar 31;18(3):e0281083. doi: 10.1371/journal.pone.0281083 (PMC10065280; doi:10.1371/journal.pone.0281083)
Supplement: S3 Table — (DOCX) [file pone.0281083.s010.docx]

**S3 Table: Results from multivariable proportional odds model indicating the odds of increased severity score (0-7) in the psychiatry, social, and youth health domains.**

| **Factors** | **Psychiatry**  OR (95% CI) | **Social**  OR (95% CI) | **Youth Health**  OR (95% CI) |
| --- | --- | --- | --- |
| **Age (per year increase)** | 1.29 (1.20, 1.39) | 1.23 (1.15, 1.33) | 1.18 (1.10, 1.26) |
| **Ethnicity** |  |  |  |
| Other terms combined | 1.22 (0.42, 3.47) | 0.73 (0.25, 2.14) | 1.10 (0.38, 3.03) |
| Asian | 0.72 (0.31, 1.66) | 1.30 (0.55, 3.12) | 0.50 (0.20, 1.19) |
| Multiethnic | 1.36 (0.77, 2.38) | 1.74 (0.97, 3.14) | 0.96 (0.55, 1.67) |
| White | - | - | - |
| **Gender** |  |  |  |
| Other terms combined | 4.19 (1.07, 16.13) | 3.76 (0.91, 15.97) | 5.03 (1.39, 18.73) |
| Girl/Young Woman | 1.31 (0.86, 2.01) | 2.03 (1.32, 3.16) | 1.08 (0.71, 1.64) |
| Boy/Young Man | - | - | - |
| **Health authority** |  |  |  |
| Fraser | 1.46 (0.86, 2.50) | 1.21 (0.69, 2.12) | 1.07 (0.62, 1.84) |
| Interior | 0.73 (0.33, 1.62) | 0.69 (0.31, 1.53) | 0.88 (0.41, 1.90) |
| Island Health | 0.81 (0.43, 1.51) | 1.36 (0.71, 2.61) | 0.74 (0.39, 1.39) |
| Northern | 0.55 (0.23, 1.30) | 0.62 (0.26, 1.50) | 0.89 (0.36, 2.13) |
| Vancouver Coastal | - | - | - |
| **Average income in area (per $5000)** | 0.90 (0.82, 0.98) | 0.91 (0.83, 0.99) | 0.91 (0.82, 0.99) |
| **School status** |  |  |  |
| Homeschool and Virtual/Remote Schooling | 0.99 (0.54, 1.81) | 1.59 (0.85, 3.01) | 2.10 (1.17, 3.77) |
| Hybrid or Part Time In-Person Schooling | 1.29 (0.69, 2.41) | 0.78 (0.41, 1.51) | 1.27 (0.68, 2.34) |
| No School or Formal Education Program | 2.30 (1.20, 4.42) | 0.92 (0.47, 1.81) | 2.10 (1.12, 3.94) |
| Summer Holiday | 0.73 (0.35, 1.52) | 1.12 (0.53, 2.37) | 0.77 (0.35, 1.61) |
| Full Time In-Person School | - | - | - |
| **Guardian unemployed** | 1.23 (0.72, 2.09) | 1.21 (0.70, 2.12) | 1.38 (0.81, 2.34) |

*OR = Odds Ratio; CI = Confidence Interval

- denotes reference
