# Supplementary material for: Evaluating the psychosocial status of BC children and youth during the COVID-19 pandemic: A MyHEARTSMAP cross-sectional study
Source: PLoS One. 2023 Mar 31;18(3):e0281083. doi: 10.1371/journal.pone.0281083 (PMC10065280; doi:10.1371/journal.pone.0281083)
Supplement: Attachment — Submitted filename: Response to Reviewers.pdf [file pone.0281083.s011.pdf]

Dear Dr. Sawsan Abuhammad
PLOS ONE Academic Editor

Re: Revision required [PONE-D-22-23103] Evaluating the psychosocial status of BC children and youth during the COVID-19 pandemic: A MyHEARTSMAP cross-sectional study

We appreciate the thoughtful review of this manuscript and the opportunity to revise the manuscript for submission. Please see below the response to reviewers and list of revisions made.

The manuscript formatting was edited to adhere to PLOS ONE guidelines including the formatting of section headings, citation brackets, and references. The specification of receiving informed verbal consent was included in our ethics statement. No other changes were made to the reference list beyond formatting.

Comments to the Author

1. Is the manuscript technically sound, and do the data support the conclusions?

The manuscript must describe a technically sound piece of scientific research with data that supports the conclusions. Experiments must have been conducted rigorously, with appropriate controls, replication, and sample sizes. The conclusions must be drawn appropriately based on the data presented.

Reviewer #1: Yes

No changes were made to the manuscript

2. Has the statistical analysis been performed appropriately and rigorously?

Reviewer #1: Yes

No changes were made to the manuscript

3. Have the authors made all data underlying the findings in their manuscript fully available?

The PLOS Data policy requires authors to make all data underlying the findings described in their manuscript fully available without restriction, with rare exception (please refer to the Data Availability Statement in the manuscript PDF file). The data should be provided as part of the manuscript or its supporting information, or deposited to a public repository. For example, in addition to summary statistics, the data points behind means, medians and variance measures should be available. If there are restrictions on publicly sharing data—e.g. participant privacy or use of data from a third party—those must be specified.

Reviewer #1: No

Data has not been made publicly available as this would violate our ethical approval due to the need for participant privacy and the potentially identifying information included in participant interviews. Data will be available for potential collaboration upon request.

4. Is the manuscript presented in an intelligible fashion and written in standard English?

PLOS ONE does not copyedit accepted manuscripts, so the language in submitted articles must be clear, correct, and unambiguous. Any typographical or grammatical errors should be corrected at revision, so please note any specific errors here.

Reviewer #1: Yes

No changes were made in the manuscript

5. Review Comments to the Author

Please use the space provided to explain your answers to the questions above. You may also include additional comments for the author, including concerns about dual publication, research ethics, or publication ethics. (Please upload your review as an attachment if it exceeds 20,000 characters)

Reviewer #1: The topic referred to children and youth however, in line 90, authors stated that "the study aims to estimate the frequency of psychosocial and health concerns of youth and their caretakers". I feel including caretakers in the aim has gone against the topic of the manuscript.

Have edited this sentence to clarify that both youth and caretakers completed assessments of the mental health of the youth only. The psychosocial status of caretakers was not assessed in this study. (line 109)

It will also be interesting to see some screenshots of the MyHEARTSMAP.

A link to the MyHEARTSMAP tool and screenshots have been included in the Supplementary Information.

I feel the OR model should make use of data from youth N= 43 and youth and guardian N= 182. I see in the manuscript that the study used data from N=424. Does that mean the study included "assessment completed by guardian only in the data analysis N= 199? I suggest that authors explicitly state the sample size included in the OR model.

I believe this mistake was due to our previous lack of clarity around caretaker assessments. The OR model included all 424 assessments. When assessments of youth mental health were

completed by both the youth and their guardian, the higher severity rating was included to increase sensitivity. The sample size of the OR model has been added. (line 290)

In table 2, the sum of youth health category 388+34+16 =438 does not add up to the total of "assessment completed by both guardian and youth" and "assessment completed by youth only" 182+43 (fig.1). Line 244 referred to 73.6% of youth which is equivalent to 312 youths. This does not add up to the total number of youths that participated in the assessment 182+42+ 225

As noted in the footnote for this table, more than one recommendation may be triggered for different questions within a domain and therefore the total number of recommendations within a domain may exceed 100%. (line 346) This note was also added to the methods to promote clarity. (line 234)

Findings make sense. I think it would be interesting to see how the use of the platform evolved and whether the users find resources, other than nurses and health care professionals, useful in improving their mental health.

We are currently analysing the data from the three-month follow-up study and preparing that work for publication so we are excited to hear that this is a matter of interest for the reviewer.

Thank you for your time and thought for this review in improving our manuscript.

Sincerely,

Melissa Woodward

Melissa Woodward

Postdoctoral Fellow
